# Person-Oriented Versus Technique-Oriented Specialties: Early Preferences and Eventual Choice

**DOI:** 10.3885/meo.2009.Res00284

**Published:** 2009-02-19

**Authors:** R. Stephen Manuel, Nicole J. Borges, Bonnie J. Jones

**Affiliations:** * Office of Admissions and Student Affairs, University of Cincinnati College of Medicine, Cincinnati, OH, USA; † Office of Academic Affairs and Department of Community Health, Boonshoft School of Medicine Wright State University, Dayton, OH, USA; ‡ Academic Affairs, University of South Florida, Sarasota-Manatee, Sarasota, FL, USA

**Keywords:** career, medical students, specialty

## Abstract

**Background:**

Students’ selection of a specialty is an important decision in their career as a physician. While distinguishing primary care physicians from non-primary care specialists has served a purpose for how medicine is practiced and managed, considering alternative ways of grouping specialties is appropriate when exploring specialty decisions.

**Purpose:**

This study explored how early specialty preferences correspond to eventual specialty choice using the person-oriented versus technique-oriented taxonomy.

**Method:**

Participants were 349 students who completed a career plan survey during the first semester of medical school and later graduated.

**Results:**

Chi-square analysis showed a statistically significant difference between students’ early preference for a person-oriented or technique-oriented specialty and the specialty they chose for their residency.

**Conclusion:**

Students with an early preference for person-oriented specialties were more likely to choose a person-oriented specialty, whereas students with an early preference for technique-oriented specialties were less likely to enter a technique-oriented specialty.

The topic of specialty choice within the field of medicine has received attention in the literature for roughly 40 years. For example, over the years several reviews of the literature on medical specialty choice have been published.^1^–^6^ The idea that specialty choice is influenced by multiple factors has been supported by various models including the one published by Bland, Meurer, and Maldonado in 1995.^3^ Among the determinants and influences related to medical student specialty choice they described were type of school, faculty composition, admissions, curriculum, students’ characteristics and values, and perceptions of specialties. Other authors^7^,^8^ support that one's experience as a medical student, students’ performance levels in certain specialty areas, as well as the students’ values, gender, and interests are among the factors influencing specialty decision making.

Despite the reviews of the literature and available models to guide inquiry concerned with medical specialty choice, as well as years of research studies examining students’ choice of specialty within the occupation of medicine, findings have yielded varying and inconsistent results regarding factors influencing choice. In addition, research suggests that though students may initially believe they know what specialty they are going to pursue upon graduation, the initial specialty is not necessarily the specialty they eventually enter.^9^ This, combined with the current state of the literature and the lack of conclusive data on choosing a specialty, suggests that continued investigation is appropriate and warranted. Though studied for decades, to date students’ selection of a specialty remains an important decision in their career as a physician.

The present study sought to contribute to the literature on specialty decision making by exploring a less understood aspect of medical student specialty decision making: how medical students’ early specialty preferences corresponded to their eventual choice of specialty. While studies of factors influencing medical specialty choice are abundant, albeit inconclusive, few studies have focused on investigating changes in specialty interests among medical students during their undergraduate medical education years. Many studies that investigated students’ early specialty interest and their later choice of specialty are dated.^10^–^11^ Additionally, the more recent studies exploring specialty choice have relied on classifications that differentiate generalists physicians (e.g., family medicine) from those who specialize in particular areas of medicine;^12^ others have categorized physicians by primary care (i.e., family medicine, internal medicine, and pediatrics) versus non-primary care specialties.^13^ Studies investigating specialty choice in general have a long history of using the primary care and non-primary care groupings. While distinguishing primary care physicians from non-primary care specialists has served a purpose for how medicine is practiced and managed, considering alternative ways of grouping specialties is appropriate when exploring specialty decisions.

The present study differs from previous investigations in that it applied a *person-oriented* versus *technique-oriented* taxonomy to understand how early specialty intentions translate into eventual choice of specialty. Person-oriented specialties refer to specialties that have more of an orientation toward people; technique-oriented describes specialties that deal more with techniques and instruments.^14^ This taxonomy focuses on what students will do in their career (i.e., working with people or performing techniques/procedures) rather than classifying students by whether they provide generalist or specialty care to patients (e.g., non-primary care versus primary care). The person-oriented versus technique-oriented approach to conceptualizing specialties was first suggested in the late 1960s,^14^,^15^ reappeared in the early 1990s,^16^ and has been used in recent studies^17^,^18^ with person-oriented specialties described as family practice, internal medicine, obstetrics/gynecology, pediatrics, physical medicine and rehabilitation, and psychiatry; specialties categorized as technique-oriented include anesthesiology, dermatology, emergency medicine, otolaryngology, pathology, radiology, and surgery. Person-orientation versus technique orientation does not imply a certain skill set, such as communication ability, but instead the functions performed on a day to day basis.

## Purpose

The purpose of the present study was to determine how medical students’ early specialty preferences corresponded to later choice of specialty using a person-oriented versus technique-oriented approach. This study is important because investigating students’ early inclination towards a technique-oriented or person-oriented field and how it corresponds to the specialty they eventually choose will help to inform and structure the medical career counseling and advising process. Results may inform career counselors and advisors about the specialty decision-making process and assist them in determining when best to begin the career exploration process with medical students. For example, is a student's first year of medical school too early to be exploring specialty interests?

## Method

**Participants** - Participants were 349 students from one midwestern medical school who completed a survey during the first semester of medical school and later graduated between 2000-2002 and 2004-2006. The survey response rate of those who completed the survey during their first three weeks of the school year was 55%. The overall response rate for students who completed the survey during their first year and then later matched in a residency prior to 2007 was 64%. Over the course of the 4 years, some students who originally participated in this study were lost to attrition, took longer than average to graduate, etc. Of the 349 students who participated, 168 (48%) were men and 181 (52%) were females. Participants included 209 (60%) Caucasians, 122 (34%) Asians, 17 (5%) under-represented minorities, and 1 (<1%) individual for whom ethnicity was not identified. The sample was demographically similar to the overall student population. With institutional review board approval, first-year medical students completed the Medical Student Career Plan Survey either during orientation or during the first 3 weeks of the semester. The survey took approximately 10 minutes to complete.

**Measure** - The Medical Student Career Plan Survey was institutionally developed to assess early student specialty interest. On the Medical Student Career Plan Survey, the students responded to the written question “If you had to choose today, what would be your top three specialty choices?” The students indicated their first, second, and third preference choice from a list of specialties provided. Gender, ethnicity, and the specialty students eventually chose were obtained from an institutional database.

**Analysis** - The first, second, and third preferences of the students and the residency match selection choice were converted using the technique and person oriented classification. Using this classification, specialty preferences of first-year medical students were compared with their specialty selection during their fourth year of medical school using a Chi-square analysis.

## Results

Results of a Chi-square analysis (*p*<.001) showed a statistically significant difference between students who indicated an initial interest in person-oriented versus technique-oriented specialties and the specialty that students selected during the fourth year of medical school. Effect sizes calculated using phi coefficients were in the medium range 19 for the first choice and in the weak range for the second and third choice. As shown in Table 1, of the 180 students who listed a person-oriented specialty as their first choice, 71% (n = 127) actually entered a person-oriented specialty and 29% (n = 53) entered a technique-oriented specialty. Students did not have to provide their first, second, and third specialty choice on the Medical Student Career Plan Survey; fifteen students did not indicate a first choice but did indicate either a second or third choice. A comparison of responses by gender (Table 2 and 3) showed that male students’ first, second, and third choices had a statistically significant relationship with their eventual specialty choices, although the effect sizes indicated that the associations were weak.^19^ Only female students’ first choices were statistically significantly related to their eventual specialty choices, with effect sizes also indicating a weak association.^19^

**Table 1.** Results of Chi-Square Analysis Comparing Students' Early Specialty Preference with their Eventual Specialty Choice

Choice	1^st^ Year area of interest	Entered a Person-Oriented Specialty	Entered a Technique-Oriented Specialty	*X*^*2*^	*p*	Effect Size (Φ)
**First**				13.02**	**<.001**	**.26**
**Person**	**180**	**127 (71%)**	**53 (29%)**			
**Technique**	**154**	**79 (51%)**	**75 (49%)**			
						
**Second**				14.70**	**<.001**	**.21**
**Person**	**176**	**126 (72%)**	**50 (28%)**			
**Technique**	**160**	**82 (51%)**	**78 (49%)**			
						
**Third**				15.37**	**<.001**	**.21**
**Person**	**141**	**104 (74%)**	**37 (26%)**			
**Technique**	**169**	**88 (52%)**	**81 (48%)**			

* Totals do not equal total sample because some students did not respond with a first, second, and third choice.

**Significant at *p* <.001 level

**Table 2.** Results of Chi-Square Analysis Comparing Students' Early Specialty Preference with their Eventual Specialty Choice for Males

Choice	1^st^ Year area of interest	Entered a Person-Oriented Specialty	Entered a Technique-Oriented Specialty	*X*^*2*^	*p*	Effect Size (Φ)
**First**				6.99**	**.008**	**.21**
**Person**	**62**	**31 (50%)**	**31 (50%)**			
**Technique**	**99**	**29 (29%)**	**70 (70%)**			
						
**Second**				3.92**	**.048**	**.155**
**Person**	**56**	**27 (48%)**	**29 (52%)**			
**Technique**	**108**	**35 (32%)**	**73 (68%)**			
						
**Third**
**Person**	**46**	**24 (52%)**	**22 (48%)**	5.144**	**.023**	**.183**
**Technique**	**107**	**35 (33%)**	**72 (67%)**			

* Totals do not equal total sample because some students did not respond with a first, second, and third choice.

**Significant at *p* <.05 level

**Table 3.** Results of Chi-Square Analysis Comparing Students' Early Specialty Preference with their Eventual Specialty Choice for Females

Choice	1^st^ Year area of interest	Entered a Person-Oriented Specialty	Entered a Technique-Oriented Specialty	*X*^*2*^	*p*	Effect Size (Φ)
**First**				7.23**	**.007**	**.204**
**Person**	**104**	**84 (81%)**	**20 (29%)**			
**Technique**	**69**	**43 (62%)**	**26 (38%)**			
						
**Second**				**2.74**	**.098**	**.126**
**Person**	**100**	**78 (78%)**	**22 (22%)**			
**Technique**	**72**	**48 (67%)**	**24 (33%)**			
						
**Third**				**2.76**	**.097**	**.132**
**Person**	**79**	**62 (78%)**	**17 (22%)**			
**Technique**	**78**	**52 (67%)**	**26 (33%)**			

* Totals do not equal total sample because some students did not respond with a first, second, and third choice.

**Significant at *p* <.05 level

Approximately 70% of students indicating a preference for person-oriented specialties during their first year of medical school, whether it was their first, second, or third choice, chose a person-oriented specialty for residency. In contrast, only about 50% of the students who initially indicated a preference for a technique-oriented specialty eventually chose a technique-oriented specialty for residency.

## Discussion

This study's findings suggest that students with an early preference for person-oriented specialties may be more likely to choose a person-oriented specialty, whereas students with early preferences for technique-oriented specialties are less likely to eventually enter a technique-oriented specialty. The study has implications for career counseling and advising students about specialty choice. Given the study's findings that about half of the students who had a preference for technique-oriented specialties actually chose a technique-oriented specialty for their residency (meaning the other half of these students chose person-oriented specialties), counselors and advisors may want to encourage students with an early interest in technique-oriented specialties to explore both person-oriented and technique-oriented specialties.

Although future investigations are warranted, it may be generally more helpful for students’ decision making to explore a variety of specialties in a particular area of medicine that is based on person orientation or technique orientation rather than exploring specialties using the primary care versus non-primary care classification. The former taxonomy focuses on what students will do in their career (i.e., working with people versus performing techniques/procedures) rather than classifying students by whether they provide generalist (primary care) versus specialty (non-primary) care to patients. Because this study showed a relationship between early preference and the specialty that students enter, students should be encouraged to begin their specialty exploration early in their medical school years. When working with students who do not yet have preferences about the areas of medicine in which they are interested, advisors can help them to determine what specialties to investigate by exploring with students whether they are more interested in working with people or performing techniques or procedures.

In this study women were less likely to move from one orientation to another; perhaps men might need to engage in a broad range of exploration. Based on this finding, further investigation regarding the stability of the person-oriented versus technique-oriented taxonomy for males and females is warranted.

In addition to the response rate limiting generalizablility of this study's findings, the current study was limited by the sample's inclusion of students from only one medical school. However, the study serves as a pilot for future, more comprehensive studies exploring the usefulness of the person-versus-technique taxonomy as it applies to specialty decision making. While this study did not ask the student how certain the student was about their first choice versus the second or third, knowing this might provide useful information and interesting results. Perhaps the 15 students in this study who did not provide a first choice were indicating that they were not sure about a specialty. This study also did not investigate students’ sub-specialty interests.

This study does not indicate why more students who expressed an early interest in a person-oriented specialty decided to pursue a person-oriented specialty. The literature suggests that a variety of factors influence specialty choice decision making: which factors were in play here? Were discrepancies in specialty preferences compared to eventual specialty choice due to a lack of knowledge regarding specialties at the beginning of medical school, or it is possible that students had different values or interests that emerged during the four years? Future studies using the person-oriented versus technique-oriented taxonomy should draw upon medical student specialty choice models described in the literature to explore the factors associated with some students pursuing their original specialty interests and some switching to different specialty areas of medicine.
